# The Influence of the COVID-19 Pandemic on Emergency Medical Services to Out-of-Hospital Cardiac Arrests in a Low-Incidence Urban City: An Observational Epidemiological Analysis

**DOI:** 10.3390/ijerph20032713

**Published:** 2023-02-03

**Authors:** Chung-Hsien Liu, Ming-Jen Tsai, Chi-Feng Hsu, Cheng-Han Tsai, Yao-Sing Su, Deng-Chuan Cai

**Affiliations:** 1 Department of Emergency Medicine, Ditmanson Medical Foundation Chia-Yi Christian Hospital, Chiayi City 600, Taiwan; 2 Graduate School of Design, National Yunlin University of Science and Technology, Yunlin 640, Taiwan; 3 Department of Emergency Medicine, Taichung Veteran’s General Hospital, Chia-Yi Branch, Chiayi City 600, Taiwan; 4 Fire Bureau, Chiayi City Government, Chiayi City 600, Taiwan

**Keywords:** emergency medical service, COVID-19 pandemic, out-of-hospital cardiac arrest

## Abstract

The Emergency Medical Services (EMS) system faced overwhelming challenges during the coronavirus disease 2019 (COVID-19) pandemic. However, further information is required to determine how the pandemic affected the EMS response and the clinical outcomes of out-of-hospital cardiac arrest (OHCA) patients in COVID-19 low-incidence cities. A retrospective study was conducted in Chiayi, Taiwan, a COVID-19 low-incidence urban city. We compared the outcomes and rescue records before (2018–2019) and during (2020–2021) the COVID-19 pandemic. A total of 567 patients before and 497 during the pandemic were enrolled. Multivariate analysis revealed that the COVID-19 pandemic had no significant influence on the achievement of return of spontaneous circulation (ROSC) and sustained ROSC but was associated with lower probabilities of survival to discharge (aOR = 0.43, 95% CI: 0.21–0.89, *p* = 0.002) and discharge with favorable neurologic outcome among OHCA patients (aOR = 0.35, 95% CI: 0.16–0.77, *p* = 0.009). Patients’ ages and OHCA locations were also discovered to be independently related to survival results. The overall impact of longer EMS rescue times on survival outcomes during the pandemic was not significant, with an exception of the specific group that experienced prolonged rescue times (total EMS time > 21 min).

## 1. Introduction

Modern cities now rely on emergency medical services (EMS), together with hospitals, to build a safety net for the healthcare of their citizens. Out-of-hospital cardiac arrest (OHCA) is a major documented cause of mortality worldwide [1]. The pre-hospital chain of survival, including emergency response activation, high-quality cardiopulmonary resuscitation (CPR), early defibrillation, advanced resuscitation, and post-cardiac arrest care and recovery is the current gold standard for OHCA patient treatment [2]. EMS response plays a critical role in treatment and is an important pre-hospital quality indicator [3].

However, EMS systems faced overwhelming challenges and increased pressure during the coronavirus disease 2019 (COVID-19) pandemic outbreak waves, causing increased EMS calls, ambulance service delays, and paramedic shortages in the affected regions or countries [4]. OHCA patients increased by 58% in Lombardy, Italy and similar patterns were reported in New York City and Paris during the early phase of the outbreak [5]. Many studies have shown similar results of significantly decreased return of spontaneous circulation (ROSC), survival rates, and discharge with favorable neurologic outcomes in OHCA patients during the pandemic [6,7,8,9]. However, most of these studies were mainly focusing on high-incidence regions or countries.

The impact of the pandemic was not only on the COVID-19-infected patients, it caused collateral mortality and morbidity among non-COVID-19 cases due to the disruption of EMS and healthcare systems, and delayed seeking of help by patients [4]. Fewer studies have investigated the influence of the pandemic on OHCA patient rescue in low-incidence areas and their results varied. Some studies demonstrated worse outcomes of ROSC and survival to discharge of OHCA patients during the pandemic even in low-incidence regions of Spain and the United States [10,11]. However, there were also studies from low-incidence regions such as West Australia and Osaka, Japan that reported that no significant differences were noted between the outcomes of OHCA patients in the pandemic compared with the pre-pandemic group [12,13]. Our research aims to focus on the influence of the pandemic on the EMS rescue of OHCA patients in a low-incidence urban city. COVID-19 has been prevalent around the globe for more than two years; therefore, we need to clarify this correlation over a longer duration.

Taiwan has implemented an effective policy of isolation, quarantine, mask-wearing, and social distancing to prevent and mitigate the outbreak of COVID-19 [14]. There were 16,508 COVID-19 cases (0.07% prevalence rate) in Taiwan from 2020 to 2021 [15]. The major outbreaks of COVID-19 were in Northern Taiwan, close to international airports, including Taipei, New Taipei, and Taoyuan City. However, Chiayi City, located in the southern part of Taiwan, had only 10 locally infected cases during 2020–2021. In this study, we aimed to determine the influence of COVID-19 on the EMS for OHCA patients in an urban city characterized by a dense population, short ambulance response time, and low incidence rate.

## 2. Materials and Methods

### 2.1. Background and Settings of Study

Our observational epidemiological analysis spanned January 2018 through December 2021. The first case of COVID-19 in Taiwan was identified in January 2020, we separated the OHCA cases into two groups according to the timeline of the pandemic: Group 1 was 2018–2019, before the pandemic, and Group 2 was 2020–2021, during the pandemic. Cardiac arrest was confirmed by the lack of signs of circulation checked by qualified rescuers.

All Emergency Medical Technicians (EMTs) had taken monthly lessons in skills assessment and quality control under the instruction of three medical directors from different hospitals during the study period. According to the resuscitation guidelines of the International Liaison Committee on Resuscitation and the American Heart Association, EMTs were trained to use laryngeal mask airways (LMAs), ball-valve-masks (BVM) to secure airways, and automated external defibrillators to convert lethal cardiac arrhythmias.

OHCA patients aged <20 years, with effective orders of do-not-resuscitate (DNR) without transfer to the hospital for any reason, and with trauma were excluded.

### 2.2. Healthcare Facilities, Pre-Hospital Rescue and OHCA Registry of the City

Chiayi City, which has 264,727 inhabitants and a size of 60.03 km^2^, has the greatest population density in southern Taiwan [16]. There are five hospitals, including one tertiary, two secondary, and two primary hospitals, that receive patients from the EMS of Chiayi City and the nearby suburb of Chiayi County.

The Chiayi City EMS system consists of a dispatch center and seven stations operated 24–7 by the fire department. Once the dispatch center receives a call for a medical emergency, a team of EMTs is dispatched from the EMS station to the nearest location.

The protocol required a duty dispatcher to ask crucial questions about cardiac arrest over the phone to verify the patient’s status. Dispatcher-assisted CPR (DACPR) or bystander CPR (BSCPR) began as soon as OHCA patients had been identified. The ambulance usually arrived within several minutes and transported the patient to the closest hospital while continuing resuscitation. All OHCA patients who initiated pre-hospital rescue were documented in the registration system of the fire department.

### 2.3. Adjustment of EMS Protocol during the COVID-19 Pandemic

Before the pandemic, EMTs’ regular personal protective equipment (PPE) included surgical masks, disposable gloves, and gowns. During the pandemic, EMTs were required to upgrade PPEs to N95 masks, goggles, fluid-resistant gowns, facial shields, and shoe covers when treating suspected or confirmed COVID-19 cases. During the pandemic, OHCA patients were considered to have suspected COVID-19 and required COVID-19 tests in the hospitals. Dispatchers were also required to ask about travel, occupation, cluster, and contact history when they received a call during the pandemic. To decrease aerosol-generating procedures, airway management of OHCA patients with either a non-rebreathing mask or a supraglottic airway connected to a high-efficiency particulate air (HEPA) filter was recommended by the Taiwan Society of Emergency Medicine [17].

### 2.4. Data Collection and Outcome Measurement

Data were acquired from the city’s OHCA registration system, according to the Utstein-style guidelines, during the study period. We collected data on patient demographics, critical time intervals, dispatcher and EMT-related variables, characteristics of arrest, pre-hospital interventions, and levels of the transferred hospital.

The periods of emergency call reception were divided into day, evening, and night. The EMS reaction time was defined as the time from the dispatch call to the EMTs’ arrival on site. EMS on-site time was calculated from the time the EMTs arrived at and left the site. The EMS traffic time was defined as the time from the EMTs leaving the site to arrival at the hospital. We also recorded the time of cardiac arrest, verified by the dispatcher; the presence of DACPR or BSCPR and the number of rescuers sent out.

Specific features, including witness to cardiac arrest and shockable arrhythmia, were also analyzed. The locations of cardiac arrest were categorized into home, institution, public space, ambulance, and others. Prehospital interventions consisted of public access defibrillation (PAD) use, number of electric shocks by PAD, ventilation support by LMAs, a mechanical CPR device use, and intravenous epinephrine injection.

The outcome measurements included any ROSC noted during resuscitation and continued ROSC ≥ 24 h as the primary outcome. Survival on discharge and discharge with favorable neurological status (Glasgow Coma Scale ≥ 13) were the secondary outcomes.

### 2.5. Statistical Methods

We assumed an odds ratio (OR) of 0.35 for accomplishing discharge with favorable neurologic outcomes in OHCA patients during the pandemic, weighed up with the pre-pandemic group, and set a rate of discharge with favorable neurologic outcomes of 5% as the baseline. We used a 5% two-tailed sample size with 80% power. To achieve statistical power, we estimated that a total of 1012 OHCA patients were required. To satisfy these criteria, we included four years of data (2018–2021).

We applied the chi-square test of independence to evaluate qualitative variables between the two groups. When dealing with quantitative variables of normal distribution, the Student’s *t*-test was employed to test the differences. The Mann–Whitney U test was used to compare differences between the two groups when the dependent variable was not normally distributed.

In order to determine the independent influence of the COVID-19 pandemic on patient outcomes, forward selection of logistic regression was applied with calibration for variables with a *p*-value < 0.1, as generated from univariate statistical tests, and the reported or potential predictive factors. The variable whether OHCA occurred during the pandemic or not was forced to be added to the regression model to obtain its independent effect. The other predictive factors for calibration included patient age [18,19], total rescue time [20,21], number of dispatched EMTs, dispatch of advanced level EMTs as EMT-Paramedics (EMTP), DACPR or BSCPR [22,23,24,25], location of arrest [26], epinephrine administered by EMTP, use of LMAs, and mechanical CPR device application [27,28]. A cutoff value of *p* < 0.05 was considered statistically significant. The statistical software JASP (version 0.16.4, open source, supported by the University of Amsterdam, Amsterdam, The Netherlands) was used to run the analysis procedures.

This study was approved by the Institutional Review Board of the Ditmanson Medical Foundation Chia-Yi Christian Hospital (CYCH-IRB 2021057).

## 3. Results

### 3.1. Demographics and Characteristics of OHCA Patients

**Figure 1:**
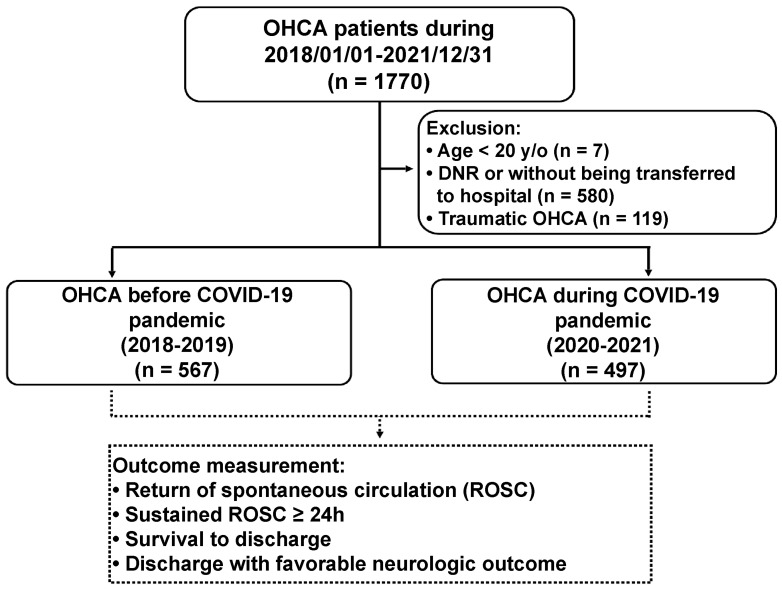
The flowchart of patient selection and grouping in our study.

There were 1770 OHCA cases collected during 2018–2021 (Figure 1). Seven patients under 20 years of age, 580 patients with valid DNR or without being transferred to the hospital, and 119 traumatic OHCA cases were excluded from the study. The number of OHCA patients enrolled before and during the COVID-19 pandemic was 567 and 497, respectively.

The clinical characteristics of the patients with OHCA before and during COVID-19 are illustrated in Table 1. The differences in age, sex, elderly percentage, and time period of emergency calls between the two groups were insignificant. However, the EMS reaction time, 4 (interquartile range [IQR] 3–5) versus 5 (IQR 3–6) minutes; on-site time, 9 (IQR 7–12) versus 10 (IQR 8–13) minutes; traffic time, 3 (IQR 2–4) versus 3 (IQR 2–5) minutes and total rescue time, 16 (IQR 14–20) versus 19 (IQR 15–22) minutes, were all significantly longer (*p* < 0.001) during the pandemic.

The ratio of DACPR or BSCPR was higher (53.19% vs. 60.16%, *p* = 0.022) during the COVID-19 pandemic. The numbers of dispatched EMTs were sometimes two people during the pandemic, compared with the standard three people before the pandemic (*p* < 0.001). The ratio of EMTP dispatches significantly increased during the pandemic (23.46% vs. 30.58%, *p* = 0.009). With regard to the characteristics of arrest, there were no differences in witnessed cardiac arrest or shockable rhythm between the two groups. However, the location of arrest differed because there were more incidents at home (75.3% vs. 77.73%) and fewer in public locations (7.41% vs. 7.09%), medical institutions (11.46% vs. 8.91%), and rescue vehicles (3.0% vs. 0.81%) during the pandemic (*p* = 0.012). In the pre-hospital treatment, LMAs (84.3% vs. 72.18%, *p* < 0.001) were used less frequently, but the use of mechanical CPR devices (29.63% vs. 78.02%, *p* < 0.001) and intravenous epinephrine (2.82% vs. 9.46%, *p* < 0.001) were significantly higher during the pandemic.

The outcome measurement revealed that there were no significant differences in any ROSC (23.8% vs. 27.9%, *p* = 0.122) or continued ROSC ≥ 24 h (16.4% vs. 19.92%, *p* = 0.137) between the pre-pandemic and the pandemic groups. However, the rate of survival to discharge (5.29% vs. 2.21%, *p* = 0.009) and discharge with favorable neurologic outcomes (5.11% vs. 1.81%, *p* = 0.004) both clearly decreased during the pandemic.

### 3.2. Influence of COVID-19 on the Primary Outcomes

To assess the association between the COVID-19 pandemic and the achievement of ROSC, a multivariate analysis (logistic regression with forward selection) was performed. After adjusting the potential predictive factors and the variables with *p* < 0.1 derived from the univariate analysis (Table 1), no significant difference in achieving ROSC was found between OHCA that occurred during the pandemic compared to OHCA that occurred during the pre-pandemic period (adjusted odds ratio [aOR] = 1.25, 95% confidence interval [CI]: 0.94–1.67, *p* = 0.129) (Table 2). The independent predictors associated with ROSC derived from the multivariate analysis were OHCA that occurred in a public place (aOR = 3.59, 95% CI: 2.22–5.83, *p* < 0.001), rescue vehicle (aOR = 4.09, 95% CI: 1.63–10.28, *p* = 0.003), and other locations (aOR = 2.28, 95% CI: 1.20–4.33, *p* = 0.011) (Table 2).

The predictive factors for continued ROSC ≥ 24 h are shown in Table 3. The same as accomplishing any ROSC, in the multivariate analysis, the COVID-19 pandemic had no significant influence in accomplishing continued ROSC ≥ 24 h (aOR = 1.32, 95% CI: 0.95–1.83, *p* = 0.102). Among the predicting factors, only the locations of OHCA were independently associated with ROSC ≥ 24 h. OHCA occurred in a public place (aOR = 4.22, 95% CI: 2.55–6.97, *p* < 0.001), in the rescue vehicle (aOR = 3.72, 95% CI: 1.43–9.71, *p* = 0.007), and in other locations (aOR = 3.51, 95% CI: 1.82–6.76, *p* < 0.001) were significantly related to ROSC ≥ 24 h.

### 3.3. Influence of COVID-19 on the Secondary Outcomes

The reported and potential predictive factors for survival to discharge are illustrated in Table 4. In contrast to achieving any ROSC and ROSC ≥ 24 h, the data showed that the COVID-19 pandemic had a negative association with survival to discharge of OHCA patients (aOR = 0.43; 95% CI: 0.21–0.89, *p* = 0.002). The other independent predictive factors associated with survival to discharge from the multivariate analysis included age (per additional year) (aOR = 0.98, 95% CI: 0.96–0.99, *p* = 0.022), and arrest in a public place (aOR = 5.67, 95% CI: 2.63–12.25, *p* < 0.001).

COVID-19 also had a negative association with favorable neurologic status on discharge (aOR = 0.35, 95% CI: 0.16–0.77, *p* = 0.009), as shown in Table 5. Moreover, each additional year of age (aOR = 0.98, 95% CI: 0.96–0.99, *p* = 0.018) and cardiac arrest in a public place (aOR = 6.20, 95% CI: 2.83–13.59, *p* < 0.001) were significantly related to discharge with a favorable neurological status.

### 3.4. Influence of the EMS Time on the Primary and Secondary Outcomes

**Figure 2:**
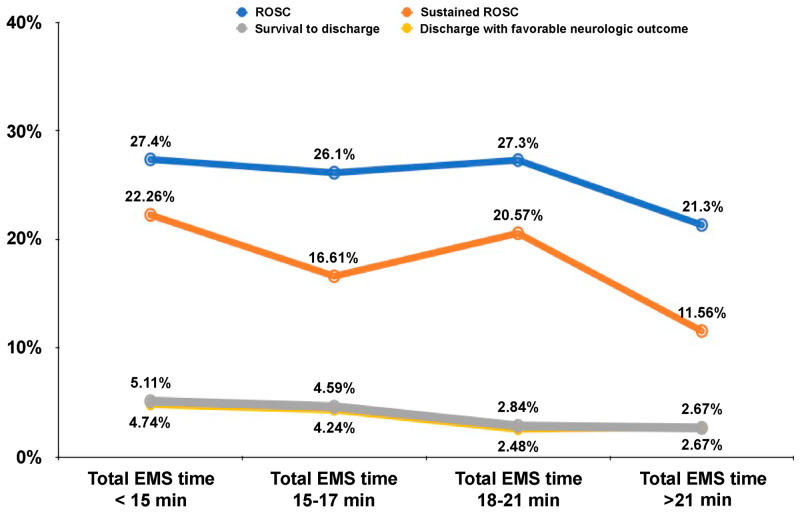
The impact of EMS rescue time on the primary and secondary outcomes.

Due to the adjusted protocol of treatment, the EMTs’ reaction time, on-site time and traffic time of OHCA patient treatment in Chiayi City were all prolonged during COVID-19. On average, it took 3 min longer for OHCA patients to be taken to hospitals during the pandemic (Table 1). The multivariate analysis (Table 2, Table 3, Table 4 and Table 5) did not show an association between each minute increase in total rescue time and the measured survival outcomes. To evaluate the overall trend of total rescue time on survival prognosis, we divided OHCA patients into four groups according to the quartile range of total rescue time (Figure 2). We found that a longer total rescue time was associated with a trend towards poorer survival to discharge and discharge with favorable neurologic outcomes after OHCA. The ratio of ROSC and continued ROSC ≥ 24 h also decreased significantly in the group of total EMS time by more than 21 min.

## 4. Discussion

Although Chiayi City had a low incidence rate of COVID-19 during 2020–2021, Taiwan implemented regulations for prevention at the same level on the entire territory. Every OHCA patient was suspected of contracting COVID-19 during the pandemic, so the EMTs followed the adjusted protocol when performing the resuscitation procedures.

According to our univariate analysis, there was an increase in mechanical CPR, intravenous epinephrine administration, and a decrease in LMAs application during the pandemic, which could be explained by fewer EMTs in ambulances, more frequent dispatching of EMT-Paramedics, and alternative use of non-rebreathing masks recommended by the adjusted protocol [17]. Many studies observed less performed BSCPR and PAD due to fear of contracting an infection during the pandemic even in low-incidence regions but the ratio of BSCPR increased and PAD use remained unchanged in our study [13,29].

Our multivariate analysis demonstrated that the independent predictive factors of the secondary outcomes included age (per additional year), locations of arrest and the COVID-19 pandemic. However, the only independent predictor of the primary outcomes was the locations of the arrest. OHCA occurred in public places had the best predictive outcomes possibly because of the patients being in better physical condition as they were able to visit public places rather than staying at home or in institutions, and the increased likelihood of early identification and initiation of the survival chain in the city [26,30]. Taiwan did not implement a large-scale lockdown due to a low incidence rate of COVID-19. Because of the recommendation for the citizens to stay home, the ratio of OHCA locations was lower in public places (7.41% vs. 7.09%, *p* = 0.012) during the pandemic. Some studies reported a 22–50% decrease in arrests in public locations due to stay-at-home orders or lockdown restrictions [12,13,30]. Therefore, the significant decrease in the number of arrests in public locations also contributed to the worse outcome of survival during the pandemic.

Although ROSC ratios were comparable between the two groups in our study, the ratios of survival to discharge and discharge with favorable outcomes were significantly lower during the pandemic. ROSC, an intermediate of OHCA survival, is directly related to the timely rescue and resuscitation quality of EMS and ED [8]. A study in Korea reported the EMS response time threshold was 11.5 min for the prehospital return of spontaneous circulation and 7.5 min for survival to discharge and favorable neurologic outcome [31].

Our results of short EMS reaction time 4 (IQR3–5) versus 5 (IQR 3–6) minutes and total EMS time 16 (IQR 14–20) versus 19 (IQR 15–22) minutes (both, *p* < 0.001) demonstrated that the rescue delays of the pandemic group are minimal but still significant. However, our multivariate analysis did not show the apparent influence of the total EMS time on ROSC and survival outcomes. The significant drop in ROSC and continued ROSC ≥ 24 h ratio in the group of total EMS time more than 21 min illustrated the concept of time threshold of OHCA rescue (Figure 2).

Based on the compensation of rescue delays to achieve comparable ROSC ratios between the two groups, we assumed that the initial resuscitation quality of the pandemic group was not inferior to the pre-pandemic group. In some studies of European countries and Japan, a physician-staffed ambulance went together with the paramedics to the primary scene to improve OHCA patient outcomes [32,33]. However, the healthcare provider shortage was a challenging issue during the pandemic. Besides, if an emergency physician works part-time in the ED and in the ambulance with frequently changing teammates, the increased risk of cross-infection should be taken into consideration during the pandemic outbreak.

In contrast to the high-incidence regions, Osaka City, Japan, Province of Padua and the Bologna area, Italy reported maintained outcomes of OHCA patients during the first wave of the pandemic, where decreased traffic time was noted particularly due to stay-at-home orders and lockdown measures [13,34,35]. In these low-incidence regions, the time spent on donning PPEs could be compensated by the shortened traffic time.

The potential factors contributing to the poorer secondary outcomes including survival to discharge and discharge with a favorable neurologic status in the pandemic group in our study are further discussed below.

First, despite the comparable outcome of ROSC, the average one-minute delay in reaction time and 3-min delay in total EMS time of the pandemic group still possibly caused irreparable damage to the brain and other vital organs that leads to poorer survival and discharge with favorable neurologic outcomes.

Second, the latter parts of the survival chain might also contribute to poorer outcomes, including advanced resuscitation in the Emergency Department (ED) and post-resuscitation care in the Intensive Care Unit (ICU). The volume of ED visits decreased significantly in Taiwan from 2020 to 2021, as a low-incidence region [36]. Theoretically, ED manpower is sufficient in the area of low incidence rates during a pandemic. However, the revised protocol of resuscitation during the pandemic for frontline providers recommended a limited number of personnel in the room, HEPA filters when using BVMs, clear plastic sheets, and externalized compression devices during advanced resuscitation [37]. These changes may have resulted in longer preparation times, more psychological strain for the caregivers, and ultimately worse outcomes for OHCA patients.

The Victoria Province of Australia, with similar features of the low-incidence rate of COVID-19, fewer OHCA cases in public locations, increased EMS response time, and comparable ROSC ratios but significantly decreased survivals during the pandemic, reported the same concern of pre-hospital treatment delays together with a change in the post-arrest care provided by the receiving hospitals [30]. The ED and ambulance crews can use in situ simulation to have better preparation and decontamination skills during the COVID-19 pandemic [38] and innovative designs of PPE, resuscitation equipment, and protocols to further decrease the preparation time and increase the effectiveness of high-quality CPR are necessary for fighting the pandemic.

Third, the revised resuscitation protocol during the pandemic also recommended the consideration of palliative care consultation and the DNR issue was widely addressed on social media especially during the first waves of the pandemic when the fatality rate was high. Furthermore, during the pandemic, hospital restrictions frequently prevented family members from visiting patients. These psychosocial aspects might have an impact on the family’s DNR decision, leading to early termination of resuscitation or withdrawal of care in the ED and ICU [30]. Due to the limitations of this observational study, these assumptions need further clarification.

The COVID-19 pandemic has lasted for more than two years and has changed the way we live our lives. Government officials need more information and statistical analysis from the healthcare frontlines to weigh up the pros and cons of implementing regulations and prevention in the post-COVID-19 era to come. Our study showed that even in a low-incidence urban city, the pandemic had a tremendous influence on the healthcare system, including the EMS.

## 5. Conclusions

Chiayi is an urban city characterized by a dense population, rapid ambulance response, and a low incidence rate of COVID-19 during the pandemic. Patients arrested in public locations had the best chance of survival to discharge and their numbers increased significantly during the pandemic. The EMS reaction time and total EMS time were prolonged slightly during the pandemic but did not show apparent influence on the results of ROSC and survival, except for a specific group that experienced prolonged rescue times (total EMS time > 21 min).

Although any ROSC and continued ROSC ≥ 24 h results were not significantly different between the pre-pandemic and pandemic groups, OHCA patients during the COVID-19 pandemic had significantly lower survival rates and poorer neurological outcomes. This suggests that while the ability of emergency medical services (EMS) to successfully revive OHCA patients during the pandemic may not have been affected, the overall outcomes for OHCA patients were worse during the pandemic. Possible explanations could be changes in protocols, disruptions of in-hospital care, and psychosocial stress to the care providers and family members during the pandemic. The results of this study are important and should be further investigated to understand the specific impact of the pandemic on OHCA patients and to improve outcomes in the future.

## Figures and Tables

**Table 1:** Specific features of OHCA patients before and during COVID-19 pandemic.

	Before COVID-19 Pandemic (n = 567)	During COVID-19 Pandemic (n = 497)	*p* Value
Demographics			
Age	76.0 (64–85)	78 (65–85)	0.545
Older adults (≥65 years)	409.0 (72.65)	367.0 (75.98)	0.219
Male gender	313 (55.40)	292 (59.00)	0.239
Time period of emergent call reception			
Day (8 AM–4 PM)	247 (43.56)	220 (44.27)	0.779
Evening (4 PM–12 PM)	168 (29.63)	153 (30.78)	
Night (12 PM–8 AM)	152 (26.81)	124 (24.95)	
Time interval of the rescue			
Reaction time (min)	4 (3–5)	5 (3–6)	<0.001
On site time (min)	9 (7–12)	10 (8–13)	<0.001
Traffic time (min)	3 (2–4)	3 (2–5)	<0.001
Total rescue time (min)	16 (14–20)	19 (15–22)	<0.001
EMS Dispatcher related			
DACPR or BSCPR	300 (53.19)	299 (60.16)	0.022
OHCA verification time (s)	55 (28–109) (n = 403)	62 (29–115) (n = 392)	0.247
DACPR beginning time (s)	172 (132–233) (n = 270)	175(131–229) (n = 291)	0.608
Numbers of EMT (Median (IQR))	3 (3–3)	3 (2–3) (n = 495)	<0.001
EMTP dispatch	133 (23.46)	152 (30.58)	0.009
Characteristics of OHCA			
Cardiac arrest witnessed	292 (51.50)	237 (47.69)	0.215
Shockable arrhythmia by AED	102 (18.25)	95 (19.55)	0.592
OHCA locations			
Home	427 (75.31)	384 (77.73)	0.012
Public place	42 (7.41)	35 (7.09)	
Institution	65 (11.46)	44 (8.91)	
Others	16 (2.82)	27 (5.47)	
On the ambulance	17 (3.00)	4 (0.81)	
Prehospital treatment			
Use of Public Access Defibrillation (PAD)	12 (2.12)	17 (3.42)	0.192
Number of PAD defibrillations	0 (0–0)	0 (0–0)	0.191
Laryngeal mask airway	478 (84.30)	358 (72.18)	<0.001
Mechanical CPR device	168 (29.63)	387 (78.02)	<0.001
Intravenous epinephrine	16 (2.82)	47 (9.46)	<0.001
Level of transferred hospital			
Primary	80 (14.11)	66 (13.66)	0.588
Secondary	301 (53.09)	244 (50.52)	
Tertiary	186 (32.80)	173 (35.82)	
Outcomes			
Any ROSC	135 (23.81)	139 (27.97)	0.122
Continued ROSC (≥24 h)	93 (16.40)	99 (19.92)	0.137
Survival to discharge	30 (5.29)	11 (2.21)	0.009
Discharge with favorable neurologic outcome	29 (5.11)	9 (1.81)	0.004

OHCA: out-of-hospital cardiac arrest; EMS: emergency medical services; EMT: emergency medical technician; EMTP: emergency medical technician-paramedic; CPR: cardiopulmonary resuscitation; DACPR: dispatcher-assisted CPR; BSCPR: bystander CPR; AED: automated external defibrillator; ROSC: return to spontaneous circulation.

**Table 2:** Predictive factors of accomplishing any ROSC.

Factors	OR (95% CI)	*p* Value	aOR (95% CI)	*p* Value
Age (per additional year)	0.99 (0.98–0.99)	0.002	-	
Total rescue time (per minute)	0.98 (0.96–1.00)	0.112	-	
Number of dispatched EMT (per person)	0.81 (0.60–1.10)	0.174	-	
Dispatch of EMTP	1.07 (0.78–1.45)	0.680	-	
BSCPR or DACPR	0.80 (0.61–1.06)	0.115	-	
OHCA locations				
Home	reference		reference	
Public place	3.71 (2.30–5.97)	<0.001	3.59 (2.22–5.83)	<0.001
Institution	0.77 (0.46–1.29)	0.320	0.81 (0.48–1.35)	0.418
Others	2.47 (1.32–4.63)	0.005	2.28 (1.20–4.33)	0.011
On the ambulance	4.58 (1.90–11.03)	<0.001	4.09 (1.63–10.28)	0.003
Laryngeal mask airway	0.96 (0.69–1.34)	0.799	-	
Mechanical CPR device	1.10 (0.84–1.45)	0.488	-	
Intravenous epinephrine	1.37 (0.79–2.37)	0.264	-	
COVID-19 pandemic	1.24 (0.94–1.64)	0.122	1.25 (0.94–1.67)	0.129

OR: odds ratio; aOR: adjusted odds ratio; CI: confidence interval.

**Table 3:** Predictive factors of accomplishing continued ROSC ≥ 24 h.

Factors	OR (95% CI)	*p* Value	aOR (95% CI)	*p* Value
Age (per additional year)	0.99 (0.98–0.99)	0.002	-	
Total rescue time (per minute)	0.96 (0.93–0.99)	0.016	-	
Number of dispatched EMT (per person)	0.93 (0.66–1.03)	0.663	-	
Dispatch of EMTP	1.02 (0.72–1.45)	0.918	-	
BSCPR or DACPR	0.88 (0.64–1.21)	0.436	-	
OHCA locations				
Home	reference		reference	
Public place	4.40 (2.69–7.20)	<0.001	4.22 (2.55–6.97)	<0.001
Institution	1.01 (0.57–1.78)	0.971	1.06 (0.60–1.87)	0.843
Others	3.48 (1.82–6.66)	<0.001	3.51 (1.82–6.76)	<0.001
On the ambulance	4.40 (1.82–10.68)	0.001	3.72 (1.43–9.71)	0.007
Laryngeal mask airway	0.89 (0.62–1.30)	0.560	-	
Mechanical CPR device	1.10 (0.80–1.51)	0.549	-	
Intravenous epinephrine	0.96 (0.49–1.87)	0.901	-	
COVID-19 pandemic	1.27 (0.93–1.73)	0.137	1.32 (0.95–1.83)	0.102

OR: odds ratio; aOR: adjusted odds ratio; CI: confidence interval.

**Table 4:** Predictive factors of accomplishing survival to discharge.

Factors	OR (95% CI)	*p* Value	aOR (95% CI)	*p* Value
Age (per additional year)	0.97 (0.96–0.99)	<0.001	0.98 (0.96–0.99)	0.022
Total rescue time (per minute)	0.93 (0.87–0.99)	0.034	-	
Number of dispatched EMT (per person)	1.81 (0.84–3.90)	0.129	-	
Dispatch of EMTP	0.76 (0.36–1.62)	0.477	-	
BSCPR or DACPR	0.98 (0.53–1.85)	0.962	-	
OHCA locations				
Home	reference		reference	
Public place	7.28 (3.51–15.14)	<0.001	5.67 (2.63–12.25)	<0.001
Institution	0.33 (0.04–2.49)	0.283	0.35 (0.05–2.63)	0.308
Others	1.75 (0.40–7.69)	0.459	1.55 (0.34–7.05)	0.574
On the ambulance	5.98 (1.64–21.79)	0.007	3.48 (0.74–16.25)	0.113
Laryngeal mask airway	0.73 (0.36–1.48)	0.385	-	
Mechanical CPR device	0.71 (0.38–1.33)	0.280	-	
Intravenous epinephrine	0.81 (0.19–3.43)	0.773	-	
COVID-19 pandemic	0.41 (0.20–0.82)	0.012	0.43 (0.21–0.89)	0.002

OR: odds ratio; aOR: adjusted odds ratio; CI: confidence interval.

**Table 5:** Predictive factors of accomplishing discharge with favorable neurologic outcome.

Factors	OR (95% CI)	*p* Value	aOR (95% CI)	*p* Value
Age (per additional year)	0.97 (0.95–0.99)	<0.001	0.98 (0.96–0.99)	0.018
Total rescue time (per minute)	0.93 (0.87–1.00)	0.055	-	
Number of dispatched EMT (per person)	1.70 (0.78–3.74)	0.184	-	
Dispatch of EMTP	0.84 (0.39–1.80)	0.661	-	
BSCPR or DACPR	1.06 (0.55–2.05)	0.855	-	
OHCA locations				
Home	reference		reference	
Public place	8.03 (3.82–16.89)	<0.001	6.20 (2.83–13.59)	<0.001
Institution	0.37 (0.05–2.76)	0.329	0.39 (0.05–2.02)	0.356
Others	1.93 (0.44–8.54)	0.386	1.73 (0.38–7.97)	0.482
On the ambulance	4.16 (0.91–19.04)	0.066	1.74 (0.22–13.91)	0.599
Laryngeal mask airway	0.87 (0.41–1.87)	0.721	-	
Mechanical CPR device	0.73 (0.38–1.41)	0.349	-	
Intravenous epinephrine	0.88 (0.21–3.74)	0.861	-	
COVID-19 pandemic	0.34 (0.16–0.73)	0.006	0.35 (0.16–0.77)	0.009

OR: odds ratio; aOR: adjusted odds ratio; CI: confidence interval.

## Data Availability

The data presented in our study were acquired from the OHCA registration system of Fire Department of Chiayi City, Taiwan and are not publicly available.

## References

[1] Myat A., Song K.J., Rea T. (2018). Out-of-hospital cardiac arrest: Current concepts. Lancet.

[2] Merchant R.M., Topjian A.A., Panchal A.R., Cheng A., Aziz K., Berg K.M. (2020). Part 1: Executive summary: 2020 American Heart Association guidelines for cardiopulmonary resuscitation and emergency cardiovascular care. Circulation.

[3] Howard I., Cameron P., Wallis L., Castren M., Lindstrom V. (2018). Quality Indicators for Evaluating Prehospital Emergency Care: A Scoping Review. Prehosp. Disaster Med..

[4] Amiry A.A.I., Maguire B.J. (2021). Emergency Medical Services (EMS) Calls During COVID-19: Early Lessons Learned for Systems Planning (A Narrative Review). Open Access Emerg. Med..

[5] Scquizzato T., D’Amico F., Rocchi M., Saracino M., Stella F., Landoni G., Zangrillo A. (2022). Impact of COVID-19 pandemic on out-of-hospital cardiac arrest system-of-care: A systematic review and meta-analysis. Prehosp. Emerg. Care.

[6] Lim Z.J., Reddy M.P., Afroz A., Billah B., Shekar K., Subramaniam A. (2020). Incidence and outcome of out-of-hospital cardiac arrests in the COVID-19 era: A systematic review and meta-analysis. Resuscitation.

[7] Bielski K., Szarpak A., Jaguszewski M.J., Kopiec T., Smereka J., Gasecka A., Wolak P., Nowak-Starz G., Chmielewski J., Rafique Z. (2021). The Influence of COVID-19 on Out-Hospital Cardiac Arrest Survival Outcomes: An Updated Systematic Review and Meta-Analysis. J. Clin. Med..

[8] Teoh S.E., Masuda Y., Tan D.J.H., Liu N., Morrison L.J., Ong M.E.H., Blewer A.L., Ho A.F.W. (2021). Impact of the COVID-19 pandemic on the epidemiology of out-of-hospital cardiac arrest: A systematic review and meta-analysis. Ann. Intensive Care.

[9] Yu J.H., Liu C.Y., Chen W.K., Yu S.H., Huang F.W., Yang M.T., Shih H.M. (2021). Impact of the COVID-19 pandemic on emergency medical service response to out-of-hospital cardiac arrests in Taiwan: A retrospective observational study. Emerg. Med. J..

[10] Ortiz F.R., Del Valle P.F., Knox E.C., Fábrega X.J., Pascual J.M.N., Rodríguez I.M., Teja-Ruiz B., Ruiz-Azpiazu J.I., Iglesias-Vázquez J.A., Echarri-Sucunza A. (2020). Influence of the COVID-19 pandemic on out-of-hospital cardiac arrest. A Spanish nationwide prospective cohort study. Resuscitation.

[11] Chan P.S., Girotra S., Tang Y., Al-Araji R., Nallamothu B.K., McNally B. (2021). Outcomes for out-of-hospital cardiac arrest in the United States during the coronavirus disease 2019 pandemic. JAMA Cardiol..

[12] Talikowska M., Ball S., Tohira H., Bailey P., Rose D., Brink D., Bray J., Finn J. (2021). No apparent effect of the COVID-19 pandemic on out-of-hospital cardiac arrest incidence and outcome in Western Australia. Resusc. Plus.

[13] Nishiyama C., Kiyohara K., Iwami T., Hayashida S., Kiguchi T., Matsuyama T., Katayama Y., Shimazu T., Kitamura T. (2021). Influence of COVID-19 pandemic on bystander interventions, emergency medical service activities, and patient outcomes in out-of-hospital cardiac arrest in Osaka City, Japan. Resusc. Plus.

[14] Summers J., Cheng H.Y., Lin H.H., Barnard L.T., Kvalsvig A., Wilson N., Baker M.G. (2020). Potential lessons from the Taiwan and New Zealand health responses to the COVID-19 pandemic. Lancet Reg. Health-West. Pac..

[15] Taiwan Center of Disease Control Statistics. https://nidss.cdc.gov.tw/nndss/Cdcwnh07?id=19CoV.

[16] Chiayi City Household Registration Service. https://household.chiayi.gov.tw/popul05/index.aspx?Parser=99,7,43.

[17] (2020). Recommendations for Pre-Hospital Respiratory Treatment during the COVID-19 Pandemic. Taiwan Emerg. Med. Bull..

[18] Hagihara A., Onozuka D., Ono J., Nagata T., Hasegawa M. (2017). Age Gender Interaction Effect on Resuscitation Outcomes in Patients with Out-of-Hospital Cardiac Arrest. Am. J. Cardiol..

[19] Andersen L.W., Bivens M.J., Giberson T., Giberson B., Mottley J.L., Gautam S., Salciccioli J.D., Cocchi M.N., McNally B., Donnino M.W. (2015). The relationship between age and outcome in out-of-hospital cardiac arrest patients. Resuscitation.

[20] Nichol G., Cobb L.A., Yin L., Maynard C., Olsufka M., Larsen J., McCoy A.M., Sayre M.R. (2016). Briefer activation time is associated with better outcomes after out-of-hospital cardiac arrest. Resuscitation.

[21] Park H.A., Ahn K.O., Lee E.J., Park J.O., On Behalf of the Korean Cardiac Arrest Research Consortium (Ko CI) (2021). Association between survival and on-scene resuscitation time in refractory out-of-hospital cardiac arrest: A cross-sectional Retrospective Study. Int. J. Environ. Res. Public Health.

[22] Riva G., Jonsson M., Ringh M., Claesson A., Djärv T., Forsberg S., Nordberg P., Rubertsson S., Rawshani A., Nord A. (2020). Survival after dispatcher-assisted cardiopulmonary resuscitation in out-of-hospital cardiac arrest. Resuscitation.

[23] Lee Y.J., Song K.J., Shin S.D., Lee S.C., Lee E.J., Ro Y.S., Ahn K.O. (2019). Dispatcher-Assisted Cardiopulmonary Resuscitation Program and Outcomes after Pediatric Out-of-Hospital Cardiac Arrest. Pediatr. Emerg. Care.

[24] Lee S.Y., Hong K.J., Shin S.D., Ro Y.S., Song K.J., Park J.H., Kong S.Y., Kim T.H., Lee S.C. (2019). The effect of dispatcher-assisted cardiopulmonary resuscitation on early defibrillation and return of spontaneous circulation with survival. Resuscitation.

[25] Siman-Tov M., Strugo R., Podolsky T., Rosenblat I., Blushtein O. (2020). Impact of dispatcher assisted CPR on ROSC rates: A National Cohort Study. Am. J. Emerg. Med..

[26] Czapla M., Zielińska M., Kubica-Cielińska A., Diakowska D., Quinn T., Karniej P. (2020). Factors associated with return of spontaneous circulation after out-of-hospital cardiac arrest in Poland: A one-year retrospective study. BMC Cardiovasc. Disord..

[27] Kim J., Kim Y.J., Han S., Choi H.J., Moon H., Kim G. (2020). Effect of Prehospital Epinephrine on Outcomes of Out-of-Hospital Cardiac Arrest: A Bayesian Network Approach. Emerg. Med. Int..

[28] Chen Y.R., Liao C.J., Huang H.C., Tsai C.H., Su Y.S., Liu C.H., Hsu C.F., Tsai M.J. (2021). The Effect of Implementing Mechanical Cardiopulmonary Resuscitation Devices on Out-of-Hospital Cardiac Arrest Patients in an Urban City of Taiwan. Int. J. Environ. Res. Public Health.

[29] Uy-Evanado A., Chugh H.S., Sargsyan A., Nakamura K., Mariani R., Hadduck K., Reinier K. (2021). Out-of-hospital cardiac arrest response and outcomes during the COVID-19 pandemic. Clin. Electrophysiol..

[30] Ball J., Nehme Z., Bernard S., Stub D., Stephenson M., Smith K. (2020). Collateral damage: Hidden impact of the COVID-19 pandemic on the out-of-hospital cardiac arrest system-of-care. Resuscitation.

[31] Lee D.W., Moon H.J., Heo N.H., KoCARC (2019). Association between ambulance response time and neurologic outcome in patients with cardiac arrest. Am. J. Emerg. Med..

[32] Sato N., Matsuyama T., Akazawa K., Nakazawa K., Hirose Y. (2019). Benefits of adding a physician-staffed ambulance to bystander-witnessed out-of-hospital cardiac arrest: A community-based, observational study in Niigata, Japan. BMJ Open.

[33] Bujak K., Nadolny K., Trzeciak P., Gałązkowski R., Ładny J.R., Gąsior M. (2022). Does the presence of physician-staffed emergency medical services improve the prognosis in out-of-hospital cardiac arrest? A propensity score matching analysis. Kardiol. Pol..

[34] Paoli A., Brischigliaro L., Scquizzato T., Favaretto A., Spagna A. (2020). Out-of-hospital cardiac arrest during the COVID-19 pandemic in the Province of Padua, Northeast Italy. Resuscitation.

[35] Semeraro F., Gamberini L., Tartaglione M., Iarussi B., Descovich C., Picoco C., Gordini G. (2020). Out-of-hospital cardiac arrest during the COVID-19 era in Bologna: System response to preserve performances. Resuscitation.

[36] Lin P.H., Su H.Y., Tsai I.T. (2022). Impact of COVID-19 Pandemic on Emergency Department Volume and Acuity in Low Incidence Area: Taiwan’s Experience in Three Hospitals. J. Acute Med..

[37] DeFilippis E.M., Ranard L.S., Berg D.D. (2020). Cardiopulmonary resuscitation during the COVID-19 pandemic: A view from trainees on the front line. Circulation.

[38] Cheng K.Y., Tu Y.C., Lu J.J., Tsai M.J., Hsu C.F. (2021). Simulation Based Ambulance and Crew Decontamination Advise During COVID-19 Pandemic. J. Acute Med..

